# Dynamic Tear Film Stability Assessed via an Open-View Double-Pass Imaging System

**DOI:** 10.1007/s44402-026-00156-7

**Published:** 2026-08-03

**Authors:** Spyridon Tsoukalas, Alexandros Pennos, Clara Bonel Pla, Anastasios Charonis, Pablo Artal, Harilaos Ginis, Dimitrios Christaras

**Affiliations:** 1 Diestia Systems PC, Athens, Greece; 2 Laboratório de Optica, University of Murcia, Murcia, Spain; 3 Research Department Oftlamosalud, Lima, Peru; 4 IMO, Barcelona, Spain; 5 Athens Vision, Athens, Greece

**Keywords:** Double-pass imaging, Dry eye, Ocular surface, Tear film

## Abstract

**Purpose:**

A novel dynamic optical metric derived from double-pass imaging called the Lacrima Stability Index (LaSI) was evaluated for the objective assessment of tear film stability.

**Methods:**

Retinal point spread functions were recorded with an open-view double-pass system at 15.4 Hz with simultaneous pupil tracking. LaSI was defined as the central PSF energy normalised by the total light exiting the eye, and the time-to-90% decay (T90) was introduced as the time for LaSI to decline by 10%.

**Results:**

In 39 eyes, LaSI traces remained stable in eyes with normal tear film stability but showed progressive decline in eyes with an unstable tear film. Using a 10-s cutoff to distinguish normal from reduced tear film stability subjects, T90 correctly classified 84.6% of eyes and showed a strong correlation with non-invasive break-up time (*r* = 0.76, *p* < 0.001). Agreement with the Ocular Surface Disease Index-6 scores was comparable for T90 and the non-invasive tear break-up time, although differences reflected the known dissociation between signs and symptoms.

**Conclusions:**

These findings suggest that LaSI provides a sensitive, time-resolved metric of tear film dynamics, with T90 serving as a clinically interpretable marker of tear film stability.

Key Points

- An open-view double-pass imaging system was introduced to monitor tear film behaviour in real time.
- The Lacrima Stability Index was defined as a dynamic metric to quantify tear film changes and directly link tear film stability with retinal image quality.
- The time-to-90% decay measure distinguished between healthy eyes and those with dry eye, showing strong agreement with established tear break-up time testing.

## Introduction

The tear film is the first refractive surface of the eye and plays a fundamental role in maintaining retinal image quality [1]. By forming a smooth optical interface, it reduces scattering and minimises aberrations. Even small instabilities in this layer can produce optical fluctuations, reduced contrast sensitivity and visual discomfort [2].

Dry eye disease (DED) is a frequently encountered multifactorial disorder arising from either deficient tear secretion or increased evaporative loss [3]. The condition compromises ocular surface integrity, producing symptoms such as irritation, redness, itching, dryness, fatigue and foreign body sensation [4]. DED is highly prevalent worldwide and has a measurable impact on visual performance in daily activities, leading to reduced work productivity [5,6]. Multiple factors contribute to tear film alterations and increase DED risk, including environmental stressors, smoking, prolonged digital screen exposure and demographics such as gender and age [7–10].

Clinical evaluation of DED typically combines subjective and objective methods. Symptom questionnaires, such as the Ocular Surface Disease Index (OSDI), assess patient-reported discomfort and functional disturbances, but do not quantify directly the optical effects of tear film instability in accordance with the Tear Film and Ocular Surface Society (TFOS) Dry eye Workshop (DEWS) III guidelines [11]. Common clinical assessments, including fluorescein tear break-up time (TBUT), Schirmer’s test and ocular surface staining, provide structural or functional information but are invasive, prone to variability and often show limited reproducibility [12–14]. Recent studies have shown that patients with tear film hyperosmolarity exhibit significant variations in intraocular light scatter over time, even when conventional signs such as TBUT and staining appear normal [15]. These findings emphasise the need for time-resolved assessments of the tear film.

Non-invasive break-up time (NIBUT) measured with Placido-disc–based systems is an established clinical technique for assessing tear film stability, offering more standardised measurements compared to TBUT [11, 16]. However, NIBUT provides only an indirect evaluation of tear film dynamics, as it relies on corneal reflection patterns rather than the retinal image quality perceived by the patient.

Optical methods have been investigated to address these limitations. Aberrometry studies have shown that higher-order aberrations increase progressively during the inter-blink period, reflecting tear film instability [17]. Similarly, double-pass (DP) imaging quantifies the retinal point spread function (PSF) and derives indices such as the Objective Scatter Index (OSI), directly linking retinal image quality to tear film performance [18,19].

In this work, we developed an optical metric that we call the Lacrima Stability Index (LaSI). It is derived from the central energy of the PSF, divided by the total amount of light entering the eye over time. Therefore, LaSI characterises temporal fluctuations and provides a dynamic description of tear film stability.

The objective of this study was to assess the validity of LaSI as a marker of tear film stability. LaSI-derived metrics were compared with NIBUT and subjective symptoms obtained from the OSDI-6 questionnaire, a short-form version of the OSDI questionnaire comprising six items. By relating high-temporal-resolution optical measurements to established clinical and patient-reported outcomes, the potential role of LaSI in both diagnostics and research was examined.

## Methods

### Experimental Setup

**Fig. 1:**
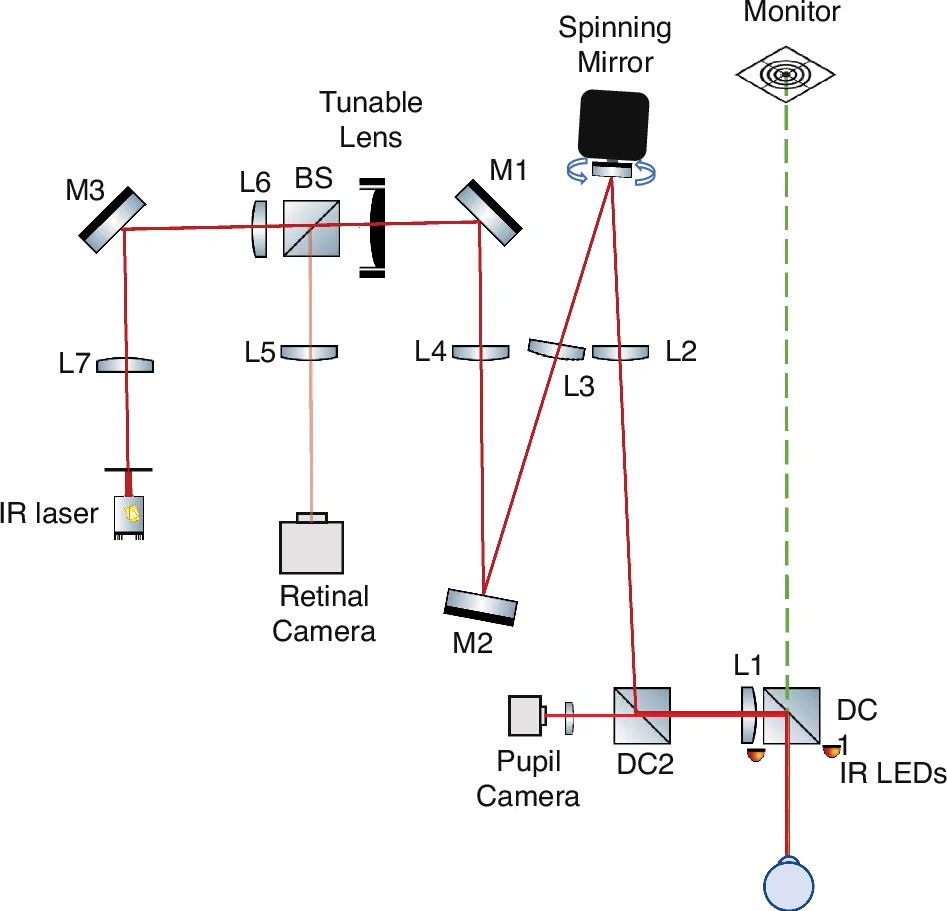
Optical layout of the open-view double-pass imaging system. BS beam splitter, DC dichroic mirrors, DV variable aperture, D1 diaphragm, IR infrared, L lenses, M mirrors.

The double-pass (DP) imaging system used in this study was configured to operate in open-view mode [20], allowing subjects to fixate binocularly on a stimulus positioned at 2 m. A collimated laser beam at 780 nm was used to generate a diffraction-limited spot on the retina through a 2 mm entrance aperture. The retinal spot PSF was then recorded through a 4 mm exit aperture by a 12 bit  complementary metal oxide semiconductor (CMOS) camera (DMM 37UX273-ML, The Imaging Source Europe GmbH, theimagingsource.com) at a sampling rate of 15.4 Hz. A spinning mirror was positioned at a pupil conjugate plane to generate a circular scan of approximately 0.5° visual angle at ~6000 rpm, effectively reducing speckle artefacts from the coherent source and averaging retinal reflections over a wider retinal area, following the approach described by Hofer et al. [21]. This configuration improved repeatability and signal quality while remaining within ISO 15004-2:2007 ophthalmic safety standards [22]. The schematic of the instrument can be seen in Fig. 1.

Prior to tear film imaging, optimal focusing was achieved using a tunable lens (Optotune EL-10-30, Optotune AG, optotune.com). The lens was scanned across a range from –10 D to +6 D in 0.1 D steps, with PSF images recorded at each setting. The lens position corresponding to the maximum central PSF intensity was selected as the best focus. Once the best focus was determined during the initial scan, the same lens position was maintained for all subsequent recordings of the retinal spot. This approach ensured not only stable focusing but also enabled a higher effective rate of image acquisition by eliminating repeated focusing steps.

**Fig. 2:**
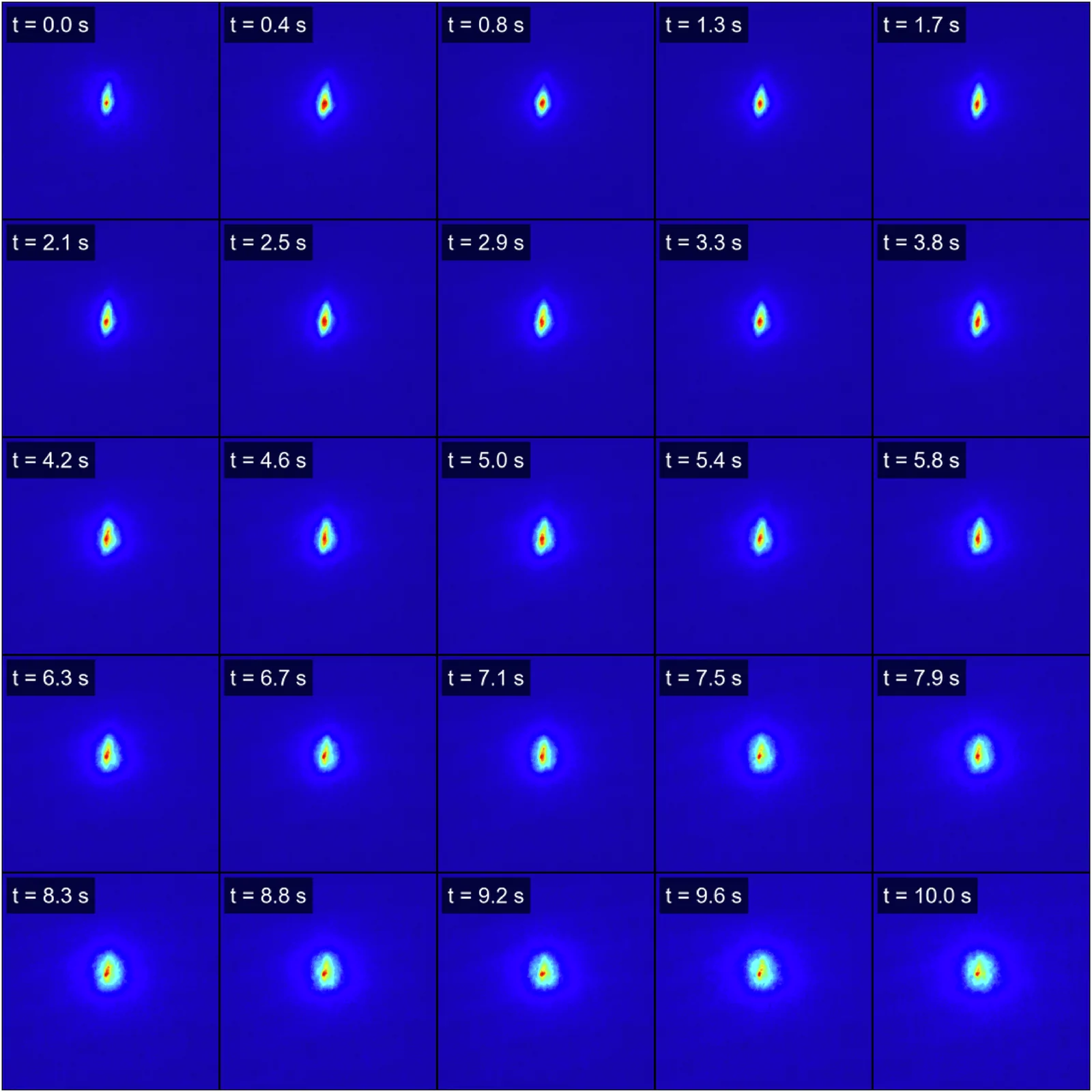
Montage of Point Spread Function (PSF) images acquired during the first 10 s after a blink (0–10 s). Progressive broadening of the PSF illustrates tear film instability.

Each measurement lasted 20 s resulting in roughly 310 images of the PSF. As an example, a montage of PSF images is shown in Fig. 2; the sequence shows a gradual broadening of the PSF, reflecting progressive optical degradation due to tear film instability.

For every retinal image of the PSF a pupil image was also captured. This was done through a dedicated near-infrared (950 nm) illumination channel and CMOS sensor (DMM 37UX287-ML, The Imaging Source Europe GmbH, theimagingsource.com) integrated into the system via a cold mirror to capture pupil frames at 15.4 Hz, the same frame rate as the retinal camera.

**Fig. 3:**
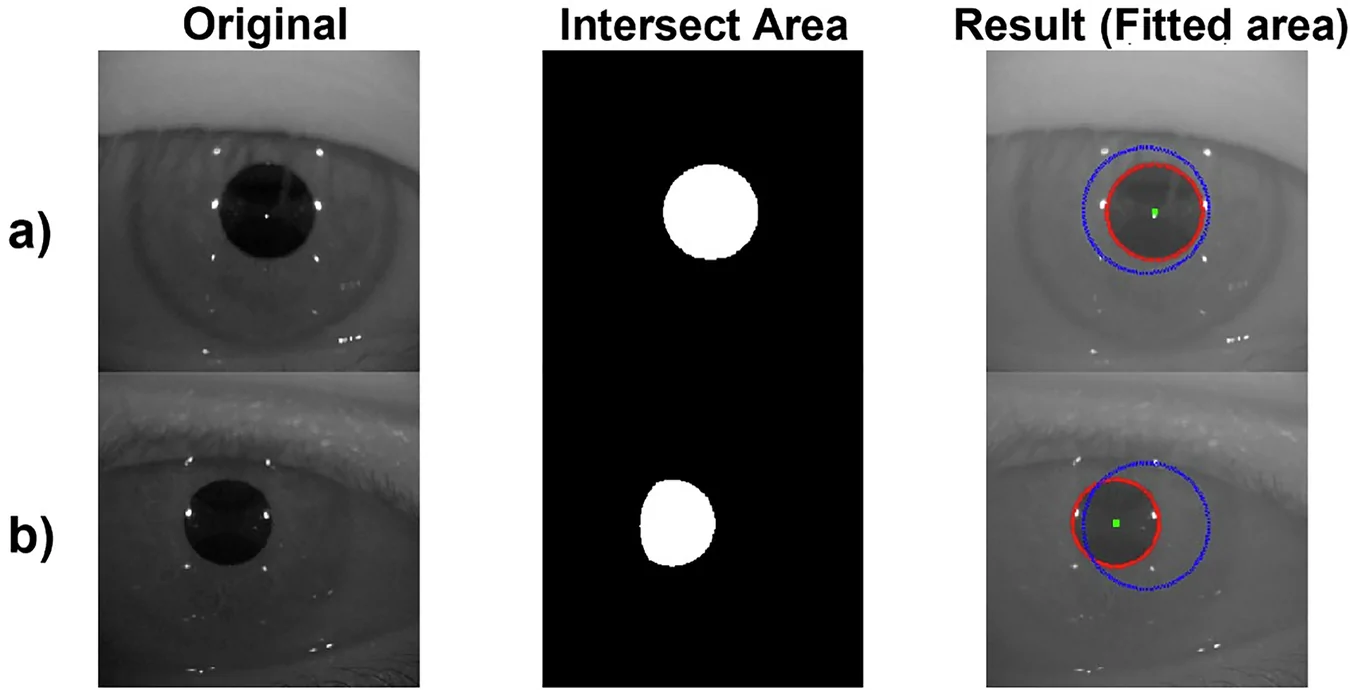
Example outputs of the pupil tracking algorithm. Left: raw near-infrared pupil images. Middle: binary masks after segmentation. Right: fitted contours (red), centroids (green) and reference discs (blue). **a** Example of pupil aligned with reference disc. **b** Example in which pupil moved outside the reference disc.

Recording of the pupil is paramount for the method; during measurement of the PSF, normal fluctuations and pupil movements will affect the amount of light entering the eye and therefore the recorded PSF. The total amount of light entering the eye depends on the size of the pupil and the intersection between the instrument’s 4 mm aperture. In order to estimate the total amount of light needed, the pupil image was first binarised using Otsu’s algorithm and the effective pupil area was found [23]. A fixed circular reference disc, centred in the pupil image (Fig. 3), was used as a stable region of interest. The overlap area between the segmented pupil mask and the reference disc was computed and stored as the intersection area A.

Intersection area A was stored for each frame and used to normalise retinal image intensity, compensating for pupil changes, eyelid intrusion and small eye movements. Frames where A approached zero or no valid pupil was detected were identified as blinks and excluded from further analysis. Quality control outputs, including segmentation overlays and pupil diameter statistics, were generated for each recording.

Optical quality was quantified using the LaSI. For each frame, the central energy (CE) of the PSF was calculated by summing pixel intensities within a 5-arcminute radius aperture centred on the PSF.

$${CE}\left(t\right)={\sum}_{\left(x,y\right)\in {R}_{5}}{I}_{t}\left(x,y\right)$$

Where CE(*t*) is the central energy at time *t*, *I*_*t*_(*x*,*y*) is the intensity of the pixel at coordinates (*x*,*y*) in time *t* and *R*₅ is the set of pixels within the 5-arcminute radius centred on the PSF.

Calibration (2 µm/pixel ≈ 0.12 arcmin/pixel) was used to convert angular dimensions to pixel units.

**Fig. 4:**
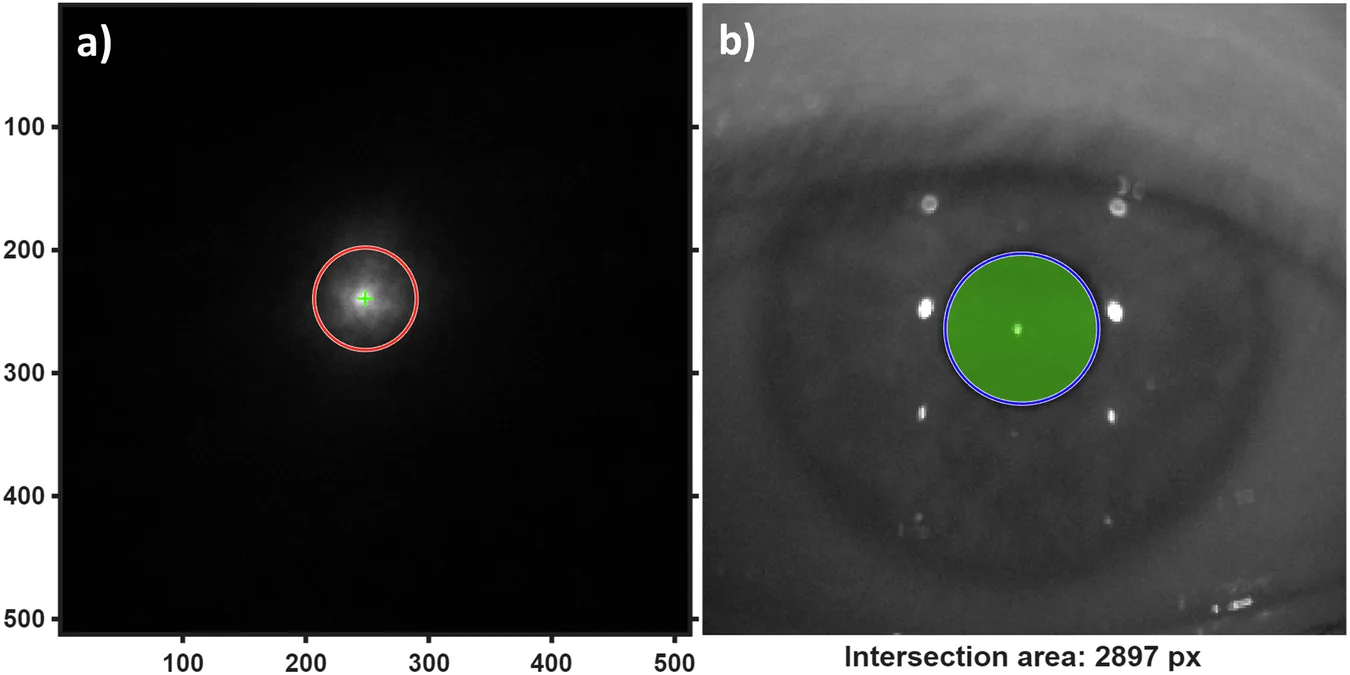
Example images used for Lacrima Stability Index (LaSI) calculation. **a** Retinal point spread function (PSF) image with the red circle indicating the limits of the 5-arcminute disc used to compute the central energy. **b** Pupil image acquired simultaneously. The green region represents the intersection area which was used to normalise the PSF intensity in the LaSI calculation.

CE was divided by A (pupil area – total light) to account for variations in pupil size and subsequently normalised to the first LaSI value (t0) to provide a relative index of temporal tear film changes with the first value set to one. Representative examples of the retinal PSF image and the simultaneously acquired pupil image used for LaSI calculation are shown in Fig. 4.

$${LaSI}\left(t\right)=\frac{{CE}\left(t\right)}{A\left(t\right)}$$

The T90 parameter was defined as the time required for LaSI to decline to 90% of baseline (a 10% reduction). Baseline was calculated as the mean LaSI during the first second of each sequence. LaSI curves were denoised with a moving-median filter using a 0.20 s window. Blinks and partial occlusions were rejected using the pupil–aperture intersection A. T90 was determined by linear interpolation between the last sample above the 90% threshold and the first sample below it. Subjects were instructed to avoid movements and gaze changes and to suppress blinking; measurements were considered valid only if T90 was reached before a blink occurred. If a blink occurred earlier, then the sequence was repeated.

### Clinical Testing

The study adhered to the Declaration of Helsinki and was approved by the Athens Vision Research Ethics Committee (Athens Vision, Athens, Greece). Testing followed a standardised protocol as specified in the case report form. Eligible participants were ≥18 years old, provided written informed consent, and were classified as normal or with suspected/diagnosed DED. Exclusion criteria included ocular surgery within the last 6 months and active ocular inflammation. Participants were instructed not to instill lubricants or eye drops for at least 30 min prior to the examination.

Demographic and background information were collected, including age, gender, best-corrected visual acuity, history of contact lens wear, ocular/systemic history and current medications. Subjective symptoms were assessed using the OSDI-6 questionnaire, with scores ≥4 considered indicative of symptomatic DED [24].

Objective tear film stability was measured with NIBUT using a Placido-disc–based topographer (Peramis, Schwind eye-tech-solutions GmbH, eye-tech-solutions.com). Measurements were performed under standardised conditions of room illumination, temperature and humidity, in accordance with TFOS DEWS III methodology. Consistent with TFOS DEWS III, NIBUT values <10 s were considered abnormal when accompanied by compatible symptoms.

All measurements were conducted in a dedicated clinical room with controlled temperature, humidity and illumination. The resulting clinical and optical data enabled direct comparison between LaSI-derived indices, NIBUT and OSDI-6 scores.

The relationship between NIBUT and T90 was evaluated using Pearson and Spearman correlation coefficients. Agreement between the objective indices (T90 and NIBUT) and OSDI-6 classification was assessed with Cohen’s κ. The discriminative performance of the LaSI-derived metrics was examined by receiver operating characteristic (ROC) analysis, with the area under the curve (AUC) calculated for T95, T90 and T85 using NIBUT <10 s as the reference criterion. Statistical analyses were performed using MATLAB (version R2025a, The MathWorks, Inc., mathworks.com).

## Results

A total of 39 eyes were included: 27 eyes from female participants (69.2%) and 12 eyes from male participants (30.8%), with a mean age 47.5 ± 19.2 years. According to the OSDI-6 cut-off (≥4), 24 eyes (61.5%) were classified as symptomatic for dry eye. Based on the Placido-disc NIBUT, subjects were divided into Low (<10 s; *n* = 28) and Normal NIBUT (≥10 s; *n* = 11) groups.

**Fig. 5:**
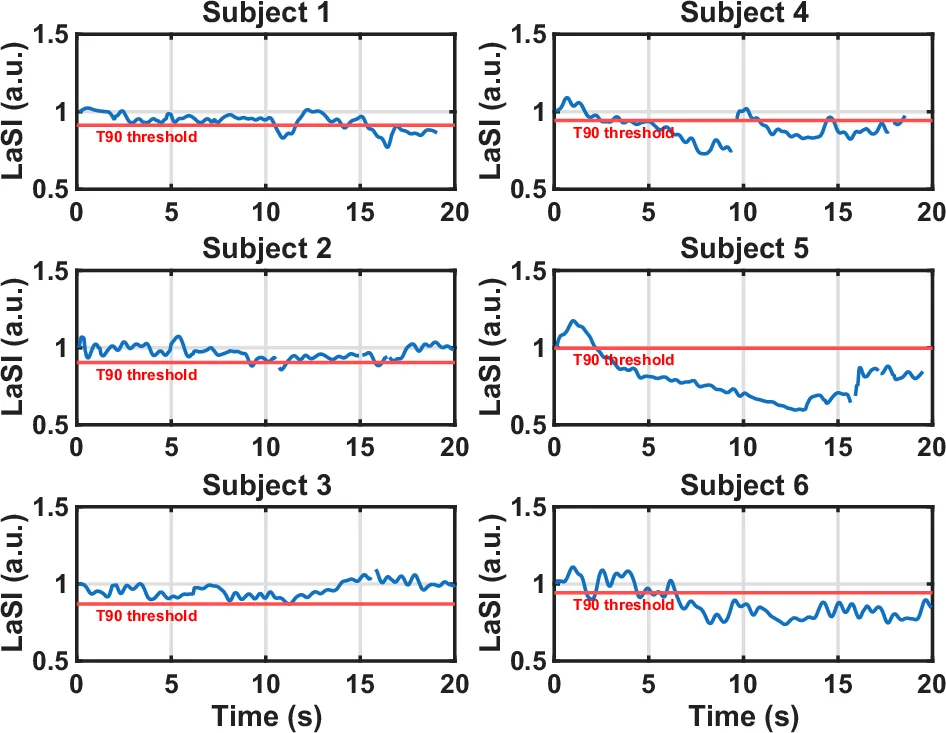
Representative Lacrima Stability Index (LaSI) traces recorded under blink-suppressed conditions over a 20-s interval. The red horizontal line indicates the individual T90 (time to 90% decay) threshold for each subject. au, arbitrary units.

Representative plots of LaSI traces obtained from the measurements are shown in Fig. 5. The curves present the temporal evolution of tear film stability, with stable curves corresponding to a normal tear film and declining curves indicating tear film instability.

**Fig. 6:**
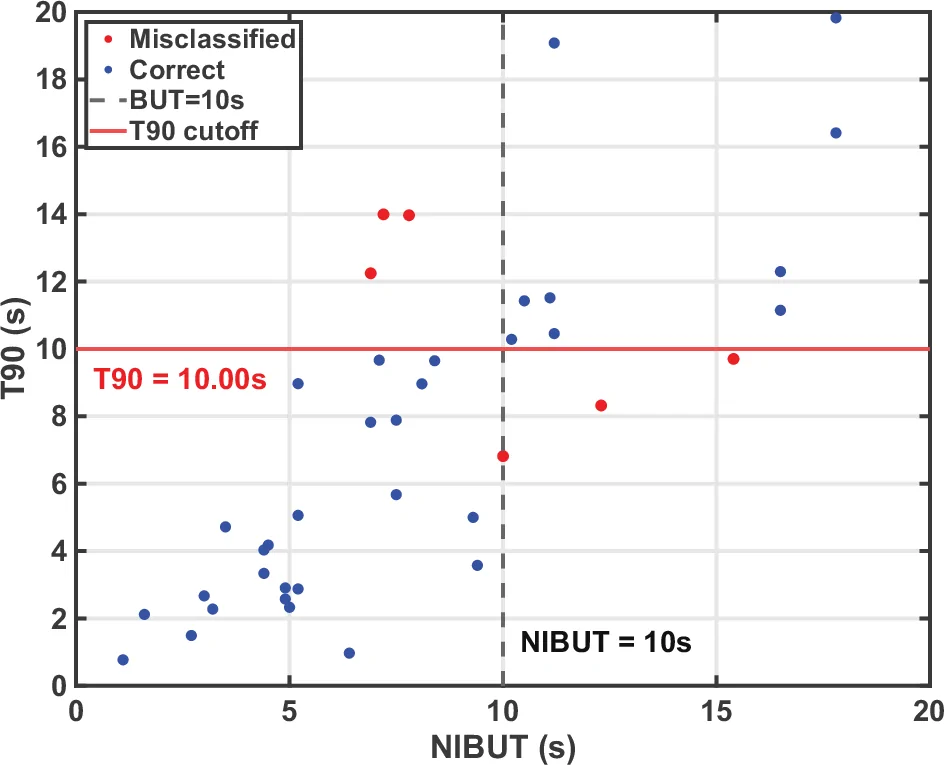
Classification of subjects based on clinical non-invasive break-up time (NIBUT) and time to 90% decay (T90) cutoff. Blue markers indicate correct classifications; red markers indicate misclassifications. The vertical dashed line marks the NIBUT cutoff of 10 s and the horizontal line marks the T90 cutoff of 10 s. BUT tear break up time.

To allow direct comparison, the same 10 s threshold was applied to the LaSI-derived metric. Using a T90 cutoff of 10 s, the method achieved an overall accuracy of 84.6%, with the classification coinciding in the two systems for 33 of 39 eyes (Fig. 6). Eyes in the Low NIBUT group exhibited significantly shorter T90 values compared with the Normal NIBUT group.

**Fig. 7:**
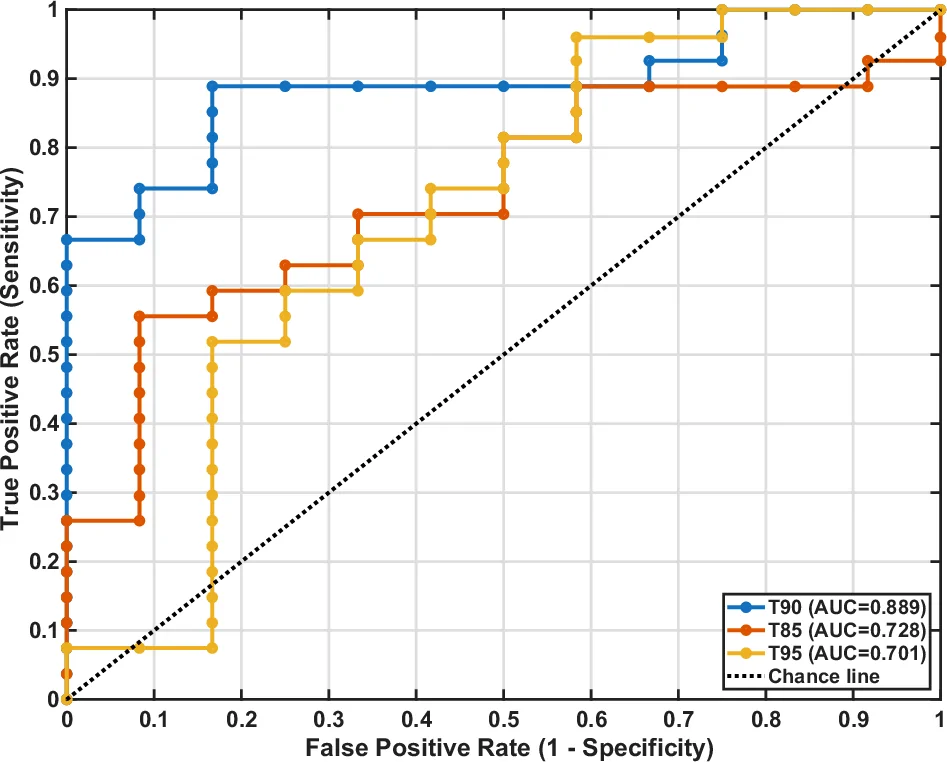
Receiver operating characteristic (ROC) analysis comparing time to 90%, 85% and 95% decay, respectively (T90, T85 and T95) for classification of tear-film instability, using non-invasive break-up time (NIBUT) <10 s as the reference criterion. True-positive rate (sensitivity) is plotted against false-positive rate (1–specificity). The dashed diagonal represents chance level. AUC area under the curve.

To determine the optimal attenuation threshold objectively, ROC analysis was performed comparing T95 (5% decay), T90 (10% decay) and T85 (15% decay), using NIBUT <10 s as the reference classification criterion. T90 demonstrated the highest discriminative performance (Area under the ROC curve (AUC) = 0.89), compared with T85 (AUC = 0.73) and T95 (AUC = 0.70), supporting the 10% attenuation threshold as the optimal cut-off for classifying tear film stability (Fig. 7).

**Fig. 8:**
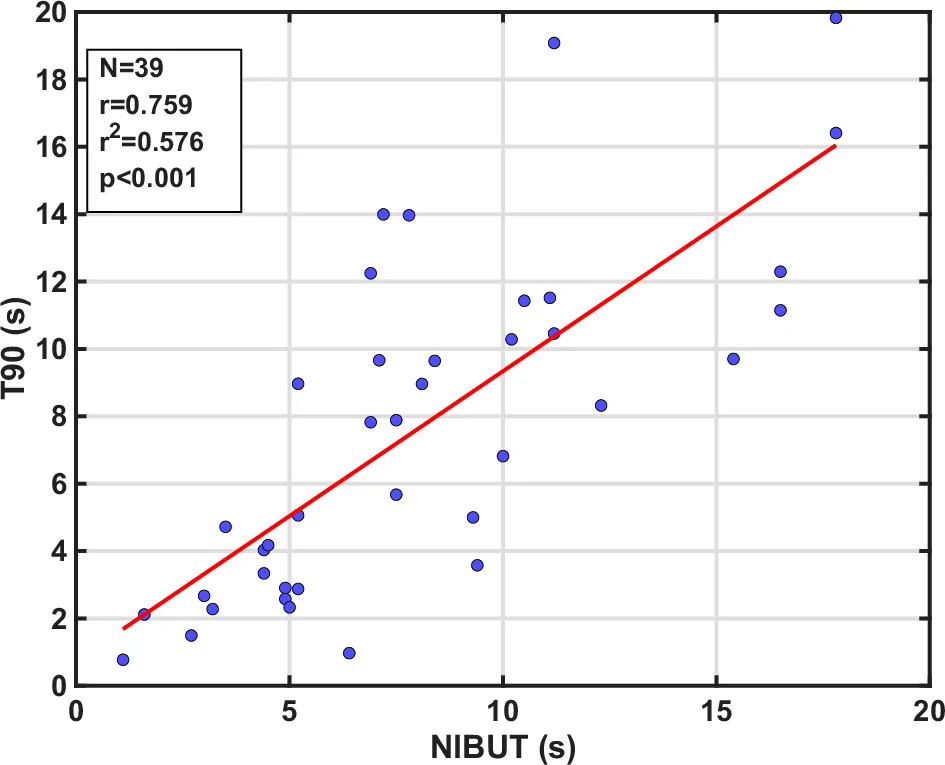
Correlation between non-invasive break-up time (NIBUT) and time to 90% decay (T90). Scatter plot showing the relationship between Placido-disc NIBUT and LaSI-derived T90 (*n* = 39). Blue dots indicate individual eyes and the red line indicates the linear regression fit. A strong positive correlation was observed (Pearson *r* = 0.76, *r*² = 0.58, *p* < 0.001, Spearman *ρ* = 0.80, *p* < 0.001).

As shown in the NIBUT–T90 scatter plot (Fig. 8), the two time-based metrics are strongly associated. Correlation analysis revealed a strong positive relationship between NIBUT and T90 (Pearson *r* = 0.76, *r*² = 0.58, *p* < 0.001, Spearman *ρ* = 0.801, *p* < 0.001), confirming that T90 reflects clinically measured tear film stability reliably.

**Fig. 9:**
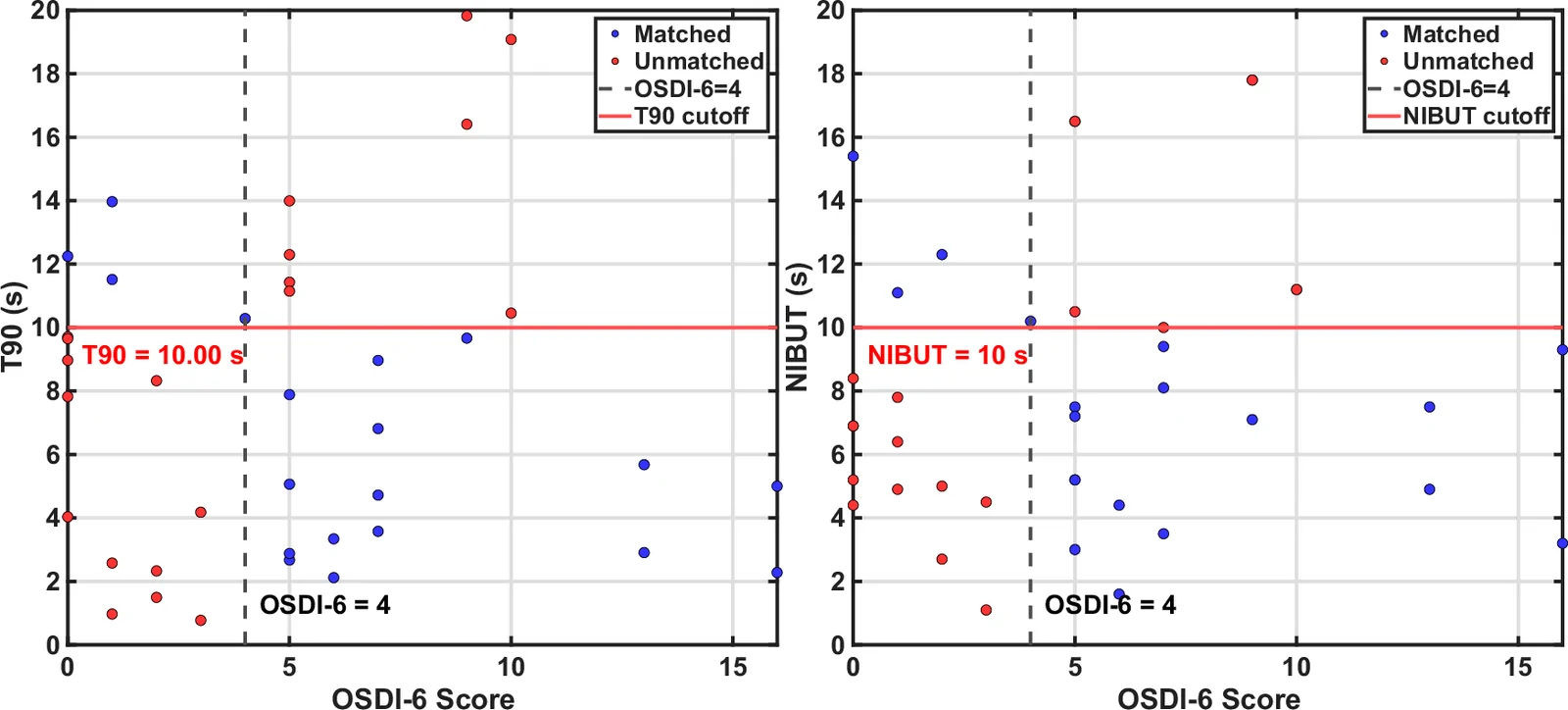
Relationship between OSDI-6 symptom scores and objective tear film stability. Left: OSDI-6 versus NIBUT. Right: OSDI-6 versus T90. Blue points indicate agreement between OSDI-6 classification (cut-off = 4) and the corresponding objective index, while red points indicate mismatches. NIBUT non-invasive break-up time, OSDI ocular surface disease index, T90 time to 90% decay.

The relationship between objective indices and subjective symptomatology was also assessed. OSDI-6 scores were compared with both NIBUT and T90 to evaluate which better reflected the symptom classification (Fig. 9). Both parameters demonstrated comparable agreement with OSDI-6 classification, with 19 matches and 20 mismatches out of 39 eyes, respectively.

## Discussion

Previous double-pass studies have reported temporal changes in retinal image quality during the inter-blink interval, showing that progressive optical degradation can serve as an indicator of tear film instability [25]. These approaches were generally performed under closed-view conditions and focused on static or averaged indices, limiting their ability to describe tear film dynamics correctly.

The LaSI offers a direct representation of tear film stability. In eyes with a normal NIBUT, the index remained stable with only minor fluctuations, whereas in eyes with low NIBUT the index showed a progressive and accelerated decline. These characteristic temporal patterns illustrate the ability of the method to capture tear film behaviour under blink-suppressed conditions.

The T90 parameter was defined as the time required for LaSI to decline to 90% of its baseline value. The selection of this threshold was supported objectively by ROC analysis comparing T95, T90 and T85, with NIBUT <10 s as the reference criterion, in which T90 demonstrated the highest performance (AUC = 0.89). The selection of this value was further supported by its good agreement with the clinical NIBUT. Using the standard 10 s cutoff, T90 correctly classified 84.6% of eyes, supporting its role as an objective measurement of tear film stability. The strong correlation between T90 and NIBUT further validates this approach.

NIBUT repeatability is known to depend on both condition and device. Same-session measurements on a multidiagnostic platform in healthy eyes showed low variability (within-subject SD ≈ 0.9 s; limits of agreement <3.5 s), indicating strong short-term repeatability [26]. In contrast, videokeratoscopy-based assessments showed significant differences between repeated trials taken minutes apart, and only fair repeatability of grading with poor inter-system agreement, implying that reproducibility across time and measurement setup is only moderate [27].

Comparison with subjective symptom scores showed that T90 and NIBUT demonstrated equivalent levels of agreement with OSDI-6 classification, with both indices yielding identical concordance counts and chance-corrected agreement (Cohen’s κ = −0.10). In 16 eyes, both indices matched the OSDI-6 results, whereas in 17 eyes these parameters were unmatched. In six eyes, only one of the two indices matched the OSDI-6 (three with T90, three with NIBUT). These findings indicate that the indices capture partly distinct aspects of tear film behaviour. The lack of a significant correlation between NIBUT and OSDI-6 scores reported previously (*r* = −0.11, *p* = 0.43) supports the dissociation between tear film integrity and subjective symptoms [26]. Chance-corrected agreement between OSDI-6 classification (OSDI-6 > 4) and tear stability classification (T90 < 10 s or NIBUT < 10 s) demonstrated minimal agreement beyond the chance level (Cohen’s κ = −0.10). Interocular asymmetry also contributed to mismatches, in line with the literature [28]. In several cases, one eye exhibited a stable tear film while the fellow eye showed instability. Since OSDI-6 reflects binocular perception, such asymmetry may result in discrepancies with monocular objective indices. This underlines the limitation of symptom questionnaires in detecting eye-specific abnormalities in tear film dynamics.

Accommodation fluctuations were examined as part of the validation of the LaSI metric. This could be addressed theoretically by performing through-focus measurements at each time-point to identify the optimal PSF. However, such an approach would reduce the sampling rate significantly and potentially compromise the detection of rapid tear film changes. In order to evaluate this, preliminary validation measurements were performed. During LaSI acquisition, the tunable lens position corresponding to best focus was recorded for each frame, allowing estimation of residual defocus over time. Defocus fluctuations during the recordings remained <0.3D and showed no systematic drift. In addition, short through-focus sequences with static focus measurements showed that a defocus change of 0.30 D produced a 1.4% reduction in central PSF energy.

Intraocular scattering also contributed to the PSF [29]. Normalisation of LaSI to the first valid frame of each sequence reduced the impact of baseline differences in scatter between eyes, ensuring that subsequent changes primarily reflected temporal variations in tear film quality. In eyes with severe cataract, scattering may dominate the PSF and limit the ability of LaSI to isolate tear film effects. This represents a potential limitation of the method that requires further evaluation in pathological cohorts with extreme cataract.

Measurements were obtained from 39 eyes, indicating that the method is suitable for assessing tear film stability. Further validation in larger and more diverse cohorts, including different age groups and a broader range of disease severity, will be important to confirm these findings and to better define the clinical potential of the approach.

An important advantage of the instrument is its open-view configuration, allowing subjects to view natural stimuli under binocular conditions in contrast to closed-view or fixation-based approaches. This allows a more realistic assessment of the tear film and enables future exploration of tear film dynamics while subjects are carrying out specific tasks, such as watching videos or reading text.

## Conclusion

The LaSI, derived from double-pass imaging, was evaluated as a dynamic and objective metric of tear film stability. Normalisation of central PSF energy to total light, the LaSI provides a robust time-resolved indicator of optical degradation. The derived T90 parameter, defined as the time to 10% decay from baseline, showed strong agreement with NIBUT, and correctly classified 84.6% of eyes at the standard 10 s threshold. Comparison with the OSDI-6 confirmed the dissociation between symptoms and signs, while the open-view configuration allowed assessment under natural viewing conditions. These results suggest that LaSI could act as a sensitive optical biomarker of tear film dynamics and support its integration into clinical diagnostics and dry eye research.

## Data Availability

Data on this paper are not publicly available, but it may be obtained from the corresponding author upon reasonable request.
